# Use of Electronic Nicotine Delivery Systems (ENDS) in China: Evidence from Citywide Representative Surveys from Five Chinese Cities in 2018

**DOI:** 10.3390/ijerph17072541

**Published:** 2020-04-08

**Authors:** Jidong Huang, Zongshuan Duan, Yu Wang, Pamela B. Redmon, Michael P. Eriksen

**Affiliations:** 1 School of Public Health, Georgia State University, Atlanta, GA 30303, USA; zduan3@student.gsu.edu (Z.D.); ywang145@student.gsu.edu (Y.W.); meriksen@gsu.edu (M.P.E.); 2 Global Health Institutes, Emory University, Atlanta, GA 30322, USA; pam.redmon@emory.edu

**Keywords:** ENDS, electronic cigarettes, E-cigarettes, China, Chinese smokers, adult smokers

## Abstract

China is the largest cigarette consuming country in the world. The emergence of electronic nicotine delivery systems (ENDS) in China may have important implications for the Chinese tobacco market. Unfortunately, research on ENDS in China, while growing, is still limited. This study was designed to examine the awareness and use of ENDS among adult urban residents in China. Data from five citywide representative surveys conducted in 2017–2018 were used. Percentages of residents who had ever heard of, ever used, or used ENDS in the past 30 days among all residents and smokers were estimated, in total and by demographic characteristics. Multiple logistic regression models were used to estimate the adjusted associations between awareness and use of ENDS and individual-level demographic characteristics and socioeconomic status (SES). Overall, 51.3% had ever heard, 4.8% ever used, and 0.9% used ENDS in the past 30 days. Men, young adults, those with high levels of education, and current smokers were more likely to be aware of and use ENDS. Among smokers, 67.8% had ever heard, 17.1% ever used, and 3.9% used ENDS in the past 30 days, respectively. Young adult smokers and smokers with high levels of education were more likely to be aware of and use ENDS. Our study results on ENDS awareness and use patterns and associated factors in China provide important evidence to inform research and policies related to ENDS manufacture, marketing, and sales in China.

## 1. Introduction

China is the largest tobacco consuming country in the world. More than 30% of the world’s cigarettes are consumed in China, predominantly by Chinese men [1,2]. According to the 2018 China Adult Tobacco Survey, 50.5% male and 2.1% female adults aged 15 and above in China were current smokers, which had not significantly changed since 2010 [3,4]. It was estimated that two million tobacco-induced deaths could occur annually in China by 2030 and three million such deaths by 2050 if the current smoking rate is not substantially reduced [5].

The Healthy China 2030 blueprint sets an ambitious goal to reduce the overall smoking rate from 27.7% in 2015 to 20% in 2030 in China, which means that China will need to help more than 75 million smokers to successfully quit smoking in the next decade [6,7]. However, a large proportion of Chinese smokers do not intend to quit smoking and smoking is still a social norm in China [8]. For example, the 2018 China Adult Tobacco Survey found that only 16.1% of current tobacco smokers intended to quit smoking in the next 12 months. This proportion has not significantly changed since 2010 [1,9]. In addition, provision of evidence-based smoking cessation services is limited, as is the access to such services in China [10]. The combination of high smoking prevalence, low cessation intention, and limited cessation services creates critical barriers to achieving the goals set forth in the Healthy China 2030 blueprint. Therefore, alternative methods to help smokers in China quit smoking are urgently needed.

Amid the urgency to reduce the toll of cigarette smoking in China, the emergence of electronic nicotine delivery systems (ENDS) added another layer of uncertainty to China’s tobacco control efforts. ENDS, also known as electronic cigarettes, or e-cigarettes, refer to “any device with a heating element that produces an aerosol from a liquid that users can inhale” [11]. The large-scale commercialization of ENDS started in the early 2000s [12]. Although the research on ENDS is growing rapidly, to date, the long-term health risks associated with using ENDS are still largely unknown. However, studies about ENDS’ short-term effects showed that the toxic chemicals and heavy metals ENDS produce could cause cellular malfunction and lung damage [13,14,15]. More recent studies indicated an elevated level of risk of cardiovascular disease among ENDS users [16]. In terms of the short-term health risks associated with ENDS use relative to cigarette smoking, a recent comprehensive review conducted by the National Academy of Science Engineering and Medicine (NASEM) concluded that the evidence is conclusive that ENDS produce fewer toxins compared to combustible cigarettes, and a complete switch from combustible cigarettes to ENDS may be beneficial to smokers’ physical health [11].

ENDS have been promoted by some as a way to minimize the harm of tobacco, rapidly saving lives [17]. However, although a complete switch to ENDS may be beneficial to individual smokers, the population health impact of ENDS will be largely determined by the evidence in three key areas: (1) the effectiveness of ENDS in helping smokers quit cigarettes; (2) their impact on smoking initiation of youth and adult nontobacco users; and (3) the health risks associated with using ENDS, both in absolute terms and in comparison with cigarettes [11,18].

Reviewing the evidence up to 2018, the NASEM report concluded that the evidence of the impact of ENDS on smoking cessation was inconclusive [11]. Since 2018, more than a dozen studies have been published on this topic. A few of these recent studies demonstrated that smoking cessation rates were higher among those who used new generations of e-cigarettes with fast nicotine delivery, particularly in combination with intensive behavioral counseling; and smoking cessation rates were also higher among those who used e-cigarettes more frequently, compared with those who only used traditional U.S. Food and Drug Administration (FDA)-approved smoking cessation products, such as nicotine gum or patch [18,19,20]. However, other recent studies did not find evidence that ENDS use was associated with higher quit rates [21,22]. To date, the evidence on the effectiveness of ENDS on increasing smoking cessation is still mixed [23,24,25], and ENDS have not been approved by FDA for smoking cessation.

Although the role ENDS play in smoking cessation is still under investigation, ENDS’ role in promoting nicotine use among youth and young adults has been well-documented. The rate of initiation of ENDS use among adolescent nonsmokers is high in developed countries [26,27]. The U.S. National Youth Tobacco Survey (NYTS) found that 27.5% of U.S. high school students reported having used ENDS products in the past 30 days in 2018, an exponential increase from 1.5% in 2011 [28]. Data from the Global Youth Tobacco Survey (GYTS) 2014 showed that about 45% of middle school students in China had heard of ENDS and 1.2% self-reported using ENDS in the past 30 days [29]. A mobile app-based survey in China found that nearly 90% of adolescents 12-18 years old had heard of e-cigarettes and 26% were users in 2015 [30].

In China, ENDS were largely unregulated until very recently. ENDS are not classified as medical devices or tobacco products, and instead are treated as consumer electronic products [31]. In August 2018, China’s State Tobacco Monopoly Administration (STMA) and the State Administration for Market Regulation (SAMR) jointly issued a circular to prohibit selling ENDS to minors (under age 18) [32]. One year later in October 2019, these two regulators issued another circular to urge shopping websites and mobile apps to remove ENDS products from their platforms [33,34]. As a result, ENDS products are no longer available on the two major Chinese e-commerce websites, jd.com and tmall.com. Other than these measures, there are no other specific policies that regulate ENDS use at the national level. Specifically, there are no laws and administration regulations that specifically address ENDS issued by the National People’s Congress (NPC, the legislative body of the central government), the Standing Committee of NPC, and the State Council. However, at the local level, a few Chinese cities have adopted measures to regulate ENDS use in public places. For example, the city of Hang Zhou, a large city in the eastern coastal area of China, has implemented policies that prohibit ENDS use in non-smoking areas [31].

Given the large smoking population and the weak tobacco control environment in China [1,6,35], ENDS could play a large role in either accelerating the tobacco epidemic, if ENDS promoted nicotine use among a new generation of Chinese youth and young adults who would otherwise not use any nicotine or tobacco products, or decelerating the tobacco toll if ENDS could help adult smokers to quit cigarettes and eventually quit all tobacco products. The net population health benefits of ENDS will be largely determined by whether the potential benefit of cessation among adult smokers could be outweighed by the costs of tobacco initiation among Chinese youth. In either scenario, it is critically important to monitor and understand the trends of ENDS use and behavioral patterns of ENDS use in China among key subpopulation groups, such as youth and adult smokers.

Unfortunately, research on ENDS awareness and/or use in China, while growing, is still limited. A recent systematic review about ENDS awareness, use, and policies in China identified 21 studies [36]. However, none of the studies reported detailed data on ENDS use among mainland Chinese adults and adult smokers, and sociodemographic correlations with ENDS use. A recent study used 2014 city-wide representative data to assess the awareness and use of e-cigarettes among urban residents in China [37]. However, given the rapidly changing tobacco and nicotine market in China, more recent data are needed to better understand the trends in ENDS use and factors associated with ENDS use. This study fills this critical gap by examining ENDS awareness and use among adult urban residents in China using the 2017/2018 citywide representative data from five large Chinese cities. The focus on urban areas was, in part, due to the higher rates of ENDS awareness and use among urban residents in China. According to the 2015 China Adult Tobacco Survey, ENDS awareness, ever use and past 30-day use were consistently higher among urban residents than among rural residents (48.6% vs. 32.1%, 4.0% vs. 2.4%, and 0.7% vs. 0.4%, respectively) [4]. Specifically, this study aims to provide estimates of ENDS awareness and use and assess factors associated with ENDS awareness and use among urban adults and adult smokers across five large Chinese cities.

## 2. Materials and Methods

### 2.1. Study Design and Survey Participants

We conducted city-wide representative household surveys in five Chinese cities—Chengdu, Chongqing, Wuhan, Xiamen, and Xi’an—from November 2017 to March 2018. This study was part of Georgia State University’s (GSU) China Tobacco-free Cities Program, which started 11 years ago [38]. In 2015, the GSU research team collaborated with the ThinkTank Research Center for Health Development, a China-based non-government organization, the China Centers for Disease Control and Prevention, and the National Health Commission (formerly National Health and Family Planning Commission) to identify a non-random sample of five metropolitan cities in China, based on population size, national influence, local government support for tobacco control efforts and stage of readiness to take action to change social norms of tobacco use in their city.

Target population was non-institutionalized adults (defined as age 15 and above at the survey time) residing in urban areas of the five cities. The survey instrument was the Tobacco Questions for Surveys (TQS). TQS was a subset of key questions from the Global Adult Tobacco Survey (GATS), which was developed by WHO and the U.S. Centers for Disease Control and Prevention (CDC) [39]. The TQS questionnaire was designed to obtain information on respondents’ background characteristics, smoking tobacco use, ENDS awareness and use, smoking cessation, secondhand smoke (SHS) exposure, tobacco economics, media exposure, and knowledge, attitudes and perceptions toward tobacco use and SHS exposure [39]. The household surveys were conducted through indoor face-to-face interviews using handheld devices to reduce measurement error. The surveys were approved by the local Institutional Review Boards (IRBs) of the municipal health department of each city, and written informed consent was obtained from all participants.

### 2.2. Sample Design and Sample Size

Three-stage cluster sampling method was applied to the select city-wide representative samples from each city based on the principles outlined in the GATS Sample Design Manual [40]. A sample size of 2000 was required for each city to meet the GATS Sample Design Manual standards of statistical quality [36]. The expected response rate was 80%, therefore, the targeted sample size was 2500 for each of the five cities. Each Ju-Wei-Hui, which is one geographically centered community for Chinese residents, was used as one primary sampling unit (PSU) [41]. At the first stage, 25 PSUs were selected from each city based on the probability proportional to size (PPS) method, and the number of households in each PSU was used as the measurement of size. At the second stage, 100 households from each PSU were randomly selected from the household sampling frame created by mapping and listing. At the third stage, one eligible adult was randomly selected from each household to participate in the survey [41].

### 2.3. Measures and Variables

#### 2.3.1. Demographic Characteristics

Demographic variables included biological sex, age, education level, and occupation status. Age was categorized into four groups: 15–24, 25–44, 45–64, and 65 years and older. Education was categorized into four levels, including primary school completed or below, junior high school completed, senior high school completed, and college degree or above. In China, the typical age range for primary school is 6–11, junior high 12–14, senior high 15–17, and college 18 and above. Occupation was categorized as “government employee, teacher, healthcare provider,” “factory, business, agriculture, and service industry employee,” and “not in the labor force,” which included the unemployed, students, homemakers, and retired. The employment categories we used in our survey were consistent with the categories used in other national level surveys in China, such as the China Global Adult Tobacco Survey [4]. “Government employee, teacher, healthcare provider” were grouped together because most schools and hospitals are government-owned in China. Those in the second category, “factory, business, agriculture, and service industry employee”, are generally considered to be working in the private sector. This grouping method also has implications related to tobacco control, as existing smoke-free policies in China are generally implemented in government buildings, schools, and hospitals [38].

#### 2.3.2. Smoking Tobacco and ENDS Use

Participants reported whether they were currently using smoking tobacco products, including cigarettes, cigars, and pipes, daily, occasionally, or not at all. Participants who were currently smoking tobacco daily and occasionally were categorized as current smokers, and participants who were not using at all were categorized as current non-smokers. Participants also reported whether they had ever heard of any type of ENDS products, whether they had ever used any ENDS product, and whether they had used any ENDS products in the past 30 days. Participants who had used ENDS in the past 30 days were defined as past 30-day ENDS users [42].

### 2.4. Data Analyses

SAS^®^ 9.4 (SAS institute, Cary, NC, USA) was used for data management and analyses. Survey procedures were performed to account for the survey’s complex sample design features [36]. Sample weights were calculated following the weighting procedure outlined in the GATS Sample Weights Manual [40]. The final weight for each respondent was calculated using the base weight as the reciprocal of the selection probability and the post-stratification adjustment for age by gender [36]. Pairwise deletion was used to handle missing values because the proportion of participants with any missing value for the key variables was less than 5% [43]. We estimated the weighted percentages and 95% confidence intervals (CIs) of urban residents who had ever heard of ENDS, who had ever used ENDS, and who used ENDS in the past 30 days, in total and by demographic characteristics, including biological sex, age, education, and occupation, for five cities overall and for each city. Rao-Scott Chi-Square tests were performed to check whether there were associations between demographic characteristics and outcome variables. Multivariate logistic regression analysis was used to estimate the adjusted odds ratios (aOR) between the measures of ENDS awareness and use across cities, controlling for individual level demographic characteristics, including age, gender, education, occupational status, and smoking status. We also estimated the awareness and use of ENDS products among current smokers only, and age, gender, education and occupation status were adjusted for multivariate logistic regression analysis.

## 3. Results

### 3.1. Demographic Characteristics and Prevalence of Tobacco Use

Overall, approximately 42% of urban residents in the five Chinese cities had a college degree or above. Nearly half of residents were in the labor force (Table A1). In addition, 23.4% were currently smoking tobacco, ranging from 22.4% in Wuhan to 25.0% in Chongqing. Most current tobacco smokers were males (93.8%), and most smokers were aged 25–44 (40.7%) and 45–64 (37.9%) (Table A2).

### 3.2. ENDS Awareness and Use among All Adult Residents

As shown in Table 1 and Table A1, more than half of all the adult residents in the five cities (51.3%) were aware of ENDS, ranging from 45.0% in Chongqing to 58.7% in Xi’an. Less than 5% (4.8%) reported having ever used ENDS at least once, ranging from 3.7% in Wuhan to 6.6% in Xi’an. Less than 1% (0.9%) reported having ever used ENDS in the past 30 days, ranging from 0.6% in Chongqing to 1.7% in Xi’an.

#### 3.2.1. ENDS Awareness among All Adult Residents

Men (59.4%) were more likely to be aware of ENDS than women (43.0%) (*p* < 0.001). Residents who were younger and with higher levels of education were more likely to be aware of ENDS, ranging from 61.1% among adults aged 15–24 to 24.5% among adults aged 65 and above (*p* < 0.001); and 61.6% among adults who had completed college or above to 21.2% among adults with primary school completed or below (*p* < 0.001). Smokers were more likely to be aware of ENDS than non-smokers (67.8% vs. 46.3%) (*p* < 0.001) (Table 1). The relationships were consistent after controlling for other demographic characteristics and smoking status; as shown in Table 2, men were significantly more likely to be aware of ENDS products compared with women (aOR = 1.4, 95% CI = 1.1–1.6, *p* = 0.001); being in younger age groups, being in higher levels of education rank, and being employed were significantly associated with higher odds of awareness of ENDS products. After controlling for demographic characteristics, current smokers were more likely to be aware of ENDS than nonsmokers (aOR = 2.3, 95% CI = 1.9–2.7, *p* < 0.001). City-specific data can be found in Tables S1 and S2.

#### 3.2.2. ENDS Ever Use among All Adult Residents

Men (8.6%) were more likely to have ever used ENDS than women (0.9%) (*p* < 0.001). Residents with higher levels of education were more likely to have ever used ENDS, ranging from 5.9% among adults who had completed college or above to 1.2% among residents with primary school completed or below (*p* < 0.001). Smokers were more likely to have ever used ENDS than nonsmokers (17.1% vs. 1.1%) (*p* < 0.001) (Table 1). After controlling for other demographic characteristics and smoking status, as shown in Table 2, men were significantly more likely to have ever used ENDS products compared to women (aOR = 2.2, 95% CI = 1.1–4.4, *p* = 0.03); residents with higher levels of education were more likely to have ever used ENDS products. After controlling for demographic characteristics, current smokers were more likely to have ever used ENDS products than nonsmokers (aOR = 15.5, 95% CI = 9.1–26.4, *p* < 0.001). City-specific data can be found in Tables S1 and S2.

#### 3.2.3. Past 30-day ENDS Use among All Adult Residents

Men (1.6%) were more likely to use ENDS in the past 30 days than women (0.3%) (*p* < 0.001). Residents who were younger and with higher levels of education were more likely to report having the used ENDS in past 30 days, ranging from 1.5% among adults aged 15–24 to 0.2% among adults aged 65 and above (*p* = 0.02); and 1.3% among adults who had completed college or above to 0.1% among adults with primary school completed or below (*p* = 0.03). Smokers were more likely to use ENDS in the past 30 days than nonsmokers (3.9% vs. 0.04%) (*p* < 0.001) (Table 1). After controlling for other demographic characteristics and smoking status, as shown in Table 2, residents between 15–24 years old were more likely to use ENDS in the past 30 days compared to residents 65 years old and above (aOR = 4.5, 95% CI = 1.1–18.7, *p* = 0.04). Residents with higher levels of education were more likely to be past 30-day users of ENDS products. City-specific data can be found in Tables S1 and S2. 

### 3.3. ENDS Awareness and Use among Current Tobacco Smokers

As shown in Table 3 and Table A2, among all the current tobacco smokers in these five cities of China, more than two-thirds (67.8%) were aware of ENDS, ranging from 56.0% in Chongqing to 78.9% in Xi’an. Approximately 17% reported having ever used ENDS at least once, ranging from 14.4% in Wuhan to 23.1% in Xi’an. Approximately 4% reported using ENDS in the past 30 days, ranging from 1.6% in Chongqing to 6.8% in Xi’an.

#### 3.3.1. ENDS Awareness among Current Tobacco Smokers

Smokers with younger age or higher levels of education were more likely to have heard of ENDS, ranging from 84.3% among smokers aged 15–24 to 36.3% among smokers aged 65 and above (*p* < 0.001), and 79.10% among smokers who had completed college or above to 36.2% among smokers with primary school completed or below (*p* < 0.001). After controlling for other demographic characteristics, as shown in Table 4, male smokers were significantly less likely to be aware of ENDS products than female smokers (aOR = 0.6, 95% CI = 0.4–1.0, *p* = 0.03). In addition, being in younger age groups and being in higher education ranks were significantly associated with higher odds of ENDS awareness. City-specific data can be found in Table S3 and S4.

#### 3.3.2. ENDS Ever Use among Current Tobacco Smokers

Smokers with younger age or higher levels of education were more likely to have ever used ENDS, ranging from 26.6% among smokers aged 15–24 to 6.6% among smokers aged 65 years and above (*p* < 0.001), and 25.5% among smokers who had completed college or above, to 3.6% among smokers with primary school completed or below (*p* < 0.001). After controlling for other demographic characteristics, as shown in Table 4, being in younger age groups and being in higher levels of education ranks were significantly associated with higher odds of ever use of ENDS products. City-specific data can be found in Table S3 and S4.

#### 3.3.3. Past 30-day ENDS Use among Current Tobacco Smokers

Smokers with younger age or higher education were more likely to have used ENDS in the past 30 days, ranging from 8.95% among smokers aged 15–24 to 1.2% among smokers aged 65 and above (*p* < 0.001), and 6.3% among smokers who had completed college or above to 0.4% among smokers with primary school completed or below (*p* = 0.0029). After controlling for other demographic characteristics, as shown in Table 4, smokers between 15–24 years old were more likely to have used ENDS products in the past 30 days compared to smokers 65 years old and above (aOR = 4.4, 95% CI = 1.1–18.7, *p* = 0.04). Smokers with higher level education were also significantly associated with higher odds of past 30-day use of ENDS products. City-specific data can be found in Table S3 and S4.

## 4. Discussion

Our study revealed several important findings regarding ENDS awareness and use among Chinese urban adults in five large Chinese cities. First, although past 30-day ENDS use was rare (0.9%, 95% CI = 0.6%–1.3%), and ever ENDS use was moderate (4.8%, 95% CI = 4.1%–5.5%) among general urban adults, awareness of ENDS was high. More than half (51.3%, 95% CI = 48.0%–54.6%) of urban adults in our study sample were aware of ENDS. Second, significant disparities in ENDS awareness and use were observed among certain subpopulation groups defined by demographics, SES, and smoking status. Specifically, rates of ENDS awareness and use were higher among smokers, men, young adults, and those with high levels of education (high school and above). Third, awareness and use of ENDS were significantly higher among adult smokers than among the general adult population across all five cities. Specifically, among adult smokers in our study sample, more than two-thirds (67.8%, 95% CI = 63.7%–72.0%) were aware of ENDS, 17.1% (95% CI = 14.5%–19.7%) had ever used ENDS, and 3.9% (95% CI = 2.6%–5.3%) had used ENDS in the 30 days prior to the survey. Fourth, significant differences in awareness and use of ENDS also existed among the subgroups within the smoking population. For example, young adult smokers and smokers with high levels of education were more likely to be aware of and use ENDS than other smokers. Finally, our results also reveal significant geographic differences in ENDS awareness and use in China. For example, rates of ENDS awareness and use were generally higher in the cities of Chengdu and Xi’an, compared with those in the cities of Chongqing, Wuhan, and Xiamen.

Our study used the 2017–2018 data from five city-wide representative household surveys and provided timely and valuable evidence to better understand behavioral patterns related to ENDS use among adult urban residents in China. To the best of our knowledge, the most recent data on ENDS use in China came from the 2018 China Global Adult Tobacco Survey (GATS), which, to date, has only released data on overall awareness and prevalence at the national level [3]. Although national level estimates were useful in understanding the aggregated trends and behavioral patterns, the overall prevalence may mask significant disparities and differential patterns of use across cities and subpopulation groups. Our study contributes to the growing ENDS research in China by providing the first evidence of disparities across Chinese cities and differential patterns of ENDS use among subpopulation groups.

The results of our study are consistent with the findings from previous studies. A recent systematic review of ENDS awareness, use, and regulations in China reported that, among adults in the studies identified, the rate of ENDS awareness ranged from 43.6% to 66.0%, the prevalence of ever use was 2.3%, and the prevalence of past 30-day use was 0.5% [31]. Similar to previous reports, our study found that the awareness of ENDS products was high, at 51.3% (95% CI = 48.0%–54.6%) among urban adults in five cities in our study. The prevalence of ever ENDS use and past 30-day ENDS use was higher in our study, 4.8% (95% CI = 4.1%–5.5%) and 0.9% (95% CI = 0.6%–1.3%), respectively. However, these numbers were not necessarily comparable since our study included only urban residents. This review also reported that the prevalence of ever ENDS use ranged from 12.8% to 13.3% among adult smokers [31], which was lower compared with our estimate (17.1%, 95% CI = 14.5%–19.7%). In addition, this review identified sex and smoking status as influencing factors of ENDS use [31,39]. Our study results revealed that, in addition to gender and smoking status, age and education were also important factors influencing ENDS awareness and use among urban residents in China. Even among smokers, age and education level were significantly associated with ENDS awareness and use. It was noteworthy that there was no significant sex difference in ENDS use among smokers in our sample. It suggests that higher rates of ENDS use among men were due to higher rates of smoking among men. But among smokers, male and female smokers were equally like to use ENDS.

China is the largest manufacturer of ENDS in the world [44], and the world’s largest producer and consumer of cigarettes [45]. Given the significant relationship between cigarette smoking and ENDS use, and the high smoking rates among Chinese men (53.2%), a close monitoring of ENDS use trends in China is warranted [3]. There are signs that ENDS use may be increasing in China. For example, while the 2015 China Adult Tobacco Survey reported 3.1% adults had ever used ENDS [4], our study results showed that 4.8% (95% CI = 4.1%–5.5%) of adults in our sample had ever used ENDS and the 2018 China Adult Tobacco Survey reported that 5.0% adults had ever used ENDS [3]. So far, ENDS use in China is still lower than that in developed countries [31]. Data in the U.S. indicate that in 2018, the prevalence of past 30-day ENDS use among US adults was 3.2% [46], higher than 0.9% (95% CI = 0.6%–1.3%) in our study. In addition, approximately 20% of smokers currently use ENDS in the U.S. [46], significantly higher than 3.9% (95% CI = 2.6%–5.3%) reported in our study. One of the reasons for low rate of ENDS use among Chinese smokers might be that the price of combustible cigarettes is low in China, and as such it doesn’t make economic sense for many smokers to switch to ENDS [47,48]. Moreover, given the current weak tobacco control policies and lack of enforcement of these policies in China, many cigarette smokers lack the incentives to quit smoking or switch to other alternative nicotine products such as ENDS [49]. As the ENDS costs decline, and stronger smoking control policies are adopted in China, such as raising cigarette excise taxes and implementing comprehensive and stronger smoke-free air policies in public places, ENDS use may increase in the future in China.

Although ENDS use among adults is relatively low in China, ENDS use seems to be higher among Chinese youth. For example, data from the Global Youth Tobacco Survey (GYTS) in 2014 showed that about 45% of middle school students in China had heard of ENDS and 1.2% self-reported past 30-day use of ENDS [29], which is higher than 0.9% (95% CI = 0.6%–1.3%) reported among adults in our study. This pattern is troubling and similar to what has been observed in the U.S., where more than a quarter of American high school students reported vaping, compared to 3.2% among adults. Concerted efforts are needed to closely monitor ENDS use among Chinese youth and ensure there will not be a youth vaping epidemic in China.

Our study has several limitations. First, our surveys were conducted in five Chinese cities and may not be generalized to other cities or rural areas in China. Second, measures of ENDS use and smoking used in this study were self-reported by participants, which may be affected by recall bias and social desirability bias [50,51]. Third, this was a cross-sectional study, and as such, unable to establish causal relationships, and the associations found in this study need to be interpreted appropriately without causal inferences. Finally, this study didn’t include youth younger than 15 years old. Future research focusing on ENDS use among Chinese youth is needed.

## 5. Conclusions

Despite these limitations, the findings of our study provided timely and important evidence to help better understand behavioral patterns related to ENDS in China, and the differential patterns of use among subpopulation groups defined by demographics, SES, and smoking status, and across geographic regions. Our study revealed that prevalence of ENDS use was low in Chinese cities, and ENDS use in China was associated with smoking status, sex, age, and education. Given the rapidly changing nature of tobacco products in China, it is important to closely monitor ENDS use in China.

## Figures and Tables

**Table 1:** Overall awareness, ever use, and past-30-day use of ENDS among adult residents in five Chinese cities in 2018, N = 10,233.

Demographic Characteristics	Awareness of ENDS		Ever Use of ENDS		Past-30-Day Use of ENDS	
	%	95% CI	%	95% CI	%	95% CI
Total	51.3	48.0–54.6	4.8	4.1–5.5	0.9	0.6–1.3
Sex						
Male	59.4	55.6–63.2	8.6	7.2–9.9	1.6	1.0–2.2
Female	43.0	39.5–46.6	0.9	0.5–1.4	0.3	0.1–0.5
Age (Years)						
15–24	61.1	55.9–66.2	5.6	3.8–7.5	1.5	0.4–2.5
25–44	59.0	55.1–62.9	5.7	4.6–6.8	1.1	0.6–1.6
45–64	44.0	40.4–47.5	4.2	3.3–5.1	0.6	0.3–1.0
65 and above	24.5	21.1–27.9	1.6	0.9–2.3	0.2	0.0–0.4
Education Level						
Primary school completed or below	21.2	16.9–25.4	1.2	0.4–2.0	0.1	0.0–0.2
Junior high school completed	41.0	37.4–44.7	4.5	3.2–5.8	0.7	0.2–1.2
Senior high school completed	53.8	49.4–58.1	4.7	3.6–5.7	1.0	0.5–1.4
College degree or above	61.6	57.4–65.9	5.9	4.8–7.0	1.3	0.6–1.9
Occupation						
Gov. employee, teacher, healthcare provider	58.6	53.3–63.9	4.1	2.7–5.5	0.6	0.2–1.0
Factory, business, service industry employee	59.8	56.0–63.6	6.3	5.0–7.5	1.3	0.8–1.9
Not in the labor force ^1^	43.9	40.3–47.5	3.8	3.0–4.6	0.7	0.3–1.2
Current Smoking Status						
Yes	67.8	63.7–72.0	17.1	14.5–19.7	3.9	2.6–5.3
No	46.3	43.1–49.6	1.1	0.7–1.5	0.0	0.0–0.1

^1^ Respondents who were not in the labor force included students, homemakers, retired, and unemployed residents either able or unable to work.

**Table 2:** Adjusted ^1^ ORs of awareness, ever use, and past-30-day use of ENDS among adult residents in five Chinese cities in 2018.

Demographic Characteristics	Awareness of ENDS		Ever Use of ENDS		Past-30-Day Use of ENDS	
	OR	95% CI	OR	95% CI	OR	95% CI
Sex						
Male	1.4	1.1–1.6	2.2	1.1–4.4	0.4	0.2–1.1
Female	Ref.		Ref.		Ref.	
Age (Years)						
15–24	2.9	2.1–3.8	2.8	1.5–5.5	4.5	1.1–18.7
25–44	2.4	1.9–3.0	1.7	1.0–3.0	2.1	0.6–7.4
45–64	1.7	1.4–2.1	1.3	0.7–2.4	1.3	0.4–4.7
65 and above	Ref.		Ref.		Ref.	
Education Level						
Primary school completed or below	Ref.		Ref.		Ref.	
Junior high school completed	2.0	1.6–2.5	2.8	1.3–6.0	6.8	1.1–41.6
Senior high school completed	2.9	2.2–3.9	2.4	1.1–5.0	7.5	1.2–45.5
College degree or above	3.9	2.8–5.3	4.4	2.1–9.2	14.3	2.9–71.5
Occupation						
Gov. employee, teacher, healthcare provider	1.2	1.0–1.5	0.8	0.5–1.2	0.5	0.2–1.5
Factory, business, service industry employee	1.3	1.1–1.5	1.1	0.8–1.5	1.1	0.6–2.0
Not in the labor force	Ref.		Ref.		Ref.	
Current Smoking Status						
Yes	2.3	1.9–2.7	15.5	9.1–26.4	198.7	52.3–754.4
No	Ref.		Ref.		Ref.	

^1^ Controlling for gender, age, education, occupation, and smoking status.

**Table 3:** Overall awareness, ever use, and past-30-day use of ENDS among adult smokers in five Chinese cities in 2018, N = 2339.

Demographic Characteristics	Awareness of ENDS		Ever Use of ENDS		Past-30-Day Use of ENDS	
	%	95% CI	%	95% CI	%	95% CI
Total	67.8	63.2–72.4	17.1	14.5–19.6	3.9	2.6–5.3
Sex						
Male	67.6	62.9–72.2	17.0	14.3–19.7	3.6	2.3–5.0
Female	71.7	64.2–79.1	18.0	8.4–27.6	8.3	1.3–15.3
Age (Years)						
15–24	84.3	77.3–91.2	26.6	18.8–34.4	9.0	2.8–15.1
25–44	75.3	70.2–80.3	20.5	16.4–24.6	4.4	2.5–6.4
45–64	59.7	53.4–66.0	11.9	8.7–15.0	2.1	1.0–3.2
65 and above	36.3	27.4–45.3	6.6	3.1–10.1	1.2	0.0–2.5
Education Level						
Primary school completed or below	36.2	25.4–47.0	3.6	0.7–6.5	0.4	0.0–1.1
Junior high school completed	60.0	52.9–67.0	14.3	9.8–18.8	2.6	0.8–4.5
Senior high school completed	69.9	64.3–75.4	14.3	11.3–17.2	3.4	1.8–5.0
College degree or above	79.1	74.0–84.2	25.5	21.1–30.0	6.3	3.1–9.6
Occupation						
Gov. employee, teacher, healthcare provider	70.2	60.9–79.4	15.7	10.6–20.9	2.4	0.6–4.2
Factory, business, service industry employee	74.3	69.4–79.2	19.4	15.8–23.0	4.9	3.0–6.8
Not in the labor force ^1^	61.3	55.9–66.6	14.9	12.1–17.7	3.4	1.4–5.4

^1^ Respondents who were not in the labor force included students, homemakers, retired, and unemployed residents either able or unable to work.

**Table 4:** Adjusted ^1^ ORs of awareness, ever use, and past-30-day use of ENDS among adult smokers in five Chinese cities in 2018.

Demographic Characteristics	Awareness of ENDS		Ever Use of ENDS		Past-30-Day Use of ENDS	
	OR	95% CI	OR	95% CI	OR	95% CI
Sex						
Male	0.6	0.4–1.0	0.8	0.4–1.6	0.4	0.1–1.0
Female	Ref.		Ref.		Ref.	
Age (Years)						
15–24	5.1	2.5–10.5	3.3	1.6–6.6	4.4	1.1–18.7
25–44	2.9	1.9–4.4	2.1	1.1–4.0	2.0	0.6–7.0
45–64	1.9	1.3–2.8	1.5	0.8–2.8	1.3	0.4–4.6
65 and above	Ref.		Ref.		Ref.	
Education Level						
Primary school completed or below	Ref.		Ref.		Ref.	
Junior high school completed	2.2	1.5–3.3	3.9	1.7–9.2	7.0	1.1–43.4
Senior high school completed	2.9	1.9–4.5	3.3	1.4–7.9	7.5	1.2–46.1
College degree or above	4.2	2.7–6.6	6.9	3.0–15.9	14.6	2.9–74.2
Occupation						
Gov. employee, teacher, healthcare provider	1.0	0.7–1.6	0.7	0.5–1.1	0.4	0.1–1.3
Factory, business, service industry employee	1.3	1.0–1.7	1.0	0.7–1.4	1.1	0.6–2.0
Not in the labor force	Ref.		Ref.		Ref.	

^1^ Controlling for gender, age, education, and occupation.

## Supplementary Materials

The following are available online at https://www.mdpi.com/1660-4601/17/7/2541/s1, Table S1: Awareness, ever use, and past-30-day use of ENDS among adult residents in five Chinese cities in 2018, Table S2: Adjusted ORs of awareness, ever use, and past-30-day use of ENDS among adult residents in five Chinese cities in 2018, Table S3: Awareness, ever use, and past-30-day use of ENDS among adult smokers in five Chinese cities in 2018, Table S4: Adjusted ORs of awareness, ever use, and past-30-day use of ENDS among adult smokers in five Chinese cities in 2018.

## Appendix A

**Table A1:** Demographic characteristics among all adult residents–TQS, 2018.

Demographic Characteristics	Chengdu (N = 1914)		Chongqing (N = 1829)		Wuhan (N = 2251)		Xiamen (N = 2116)		Xi’an (N = 2123)		Overall (N = 10233)	
	n	%	n	%	n	%	n	%	n	%	n	%
*Sex*												
Male	876	49.2	837	51.4	1158	50.6	995	52.2	1031	50.9	4897	50.8
Female	1038	50.8	992	48.6	1093	49.4	1121	47.8	1092	49.1	5336	49.3
*Age (Years)*												
15–24	188	20.4	173	18.2	157	12.3	167	26.1	155	26.1	840	20.4
25–44	787	41.2	601	36.0	838	37.6	1069	48.6	829	38.0	4124	39.4
45–64	576	27.7	728	34.9	796	35.7	660	20.0	779	26.4	3539	29.7
65 and above	363	10.6	327	11.0	460	14.4	220	5.3	360	9.5	1730	10.5
*Education Level*												
Primary school completed or below	218	8.8	285	12.1	225	7.9	452	14.5	145	5.0	1325	9.4
Junior high school completed	394	19.5	492	23.8	522	22.8	493	23.6	364	13.9	2265	20.5
Senior high school completed	453	23.6	462	27.8	721	33.2	491	26.1	694	30.6	2821	28.2
College degree or above	838	48.1	579	36.3	763	36.1	676	35.8	903	50.5	3759	42.0
*Occupation*												
Gov. employee, teacher, healthcare provider	219	12.5	207	13.2	201	9.4	205	9.3	248	11.2	1080	11.5
Factory, business, service industry employee	747	43.5	468	28.0	765	38.5	1065	50.3	750	35.9	3795	37.8
Not in the labor force ^1^	936	44.0	1105	58.9	1262	52.0	842	40.3	1086	52.9	5231	50.7
Current Smoking Status												
Yes	414	22.6	399	25.0	521	22.4	490	23.3	515	23.0	2339	23.4
No	1499	77.4	1423	75.0	1730	77.6	1625	76.7	1608	77.0	7885	76.6
Aware of ENDS	968	55.9	736	45.0	968	46.7	976	49.9	1146	58.7	4794	51.3
Ever use of ENDS	90	4.6	69	4.4	81	3.7	73	4.3	127	6.6	440	4.8
Use ENDS in past 30 days	15	0.7	10	0.6	25	1.0	18	0.9	25	1.7	93	0.9

^1^ Respondents who were not in the labor force included students, homemakers, retired, and unemployed residents either able or unable to work.

**Table A2:** Demographic characteristics among adult tobacco smokers–TQS, 2018.

Demographic Characteristics	Chengdu (N = 414)		Chongqing (N = 399)		Wuhan (N = 521)		Xiamen (N = 490)		Xi’an (N = 515)		Overall (N = 2339)	
	n	%	n	%	n	%	n	%	n	%	n	%
*Sex*												
Male	365	89.9	371	93.5	497	95.1	470	97.1	496	95.4	2199	93.8
Female	49	10.1	28	6.5	24	4.9	20	2.9	19	4.6	140	6.2
*Age (Years)*												
15–24	31	14.0	37	14.3	25	7.3	26	15.0	26	19.0	145	14.1
25–44	189	45.5	125	36.1	186	35.6	235	52.9	205	38.6	940	40.7
45–64	147	34.7	189	41.9	229	45.1	184	26.4	237	37.3	986	37.9
65 and above	47	5.8	48	7.8	81	12.0	45	5.8	47	5.1	268	7.2
*Education Level*												
Primary school completed or below	33	6.5	49	10.1	44	7.2	106	16.8	23	5.2	255	8.6
Junior high school completed	99	20.0	123	28.0	112	22.4	167	34.7	84	14.4	585	23.2
Senior high school completed	113	29.6	120	31.9	221	42.9	119	26.7	197	38.5	770	33.9
College degree or above	168	43.9	104	30.0	139	27.5	97	21.8	208	41.9	716	34.2
*Occupation*												
Gov. employee, teacher, healthcare provider	44	12.1	33	10.4	41	7.6	53	12.1	59	10.7	230	10.6
Factory, business, service industry employee	201	53.5	117	31.0	207	42.8	264	52.1	230	45.8	1019	43.8
Not in the labor force ^1^	167	34.4	237	58.6	272	49.6	172	35.7	215	43.6	1063	45.7
Aware of ENDS	306	75.5	215	56.0	333	65.3	300	65.2	388	78.9	1542	67.8
Ever use of ENDS	74	17.2	57	14.7	74	14.4	63	15.5	110	23.1	378	17.1
Use ENDS in past 30 days	15	3.2	10	2.4	24	4.0	16	3.3	24	6.9	89	3.9

^1^ Respondents who were not in the labor force included students, homemakers, retired, and unemployed residents either able or unable to work.

## References

[1] Yang G., Wang Y., Wu Y., Yang J., Wan X. (2015). The road to effective tobacco control in China. Lancet.

[2] World Health Organization, Research for International Tobacco Control (2008). WHO Report on the Global Tobacco Epidemic, 2008: The MPOWER Package.

[3] 2018 China Global Adult Tobacco Survey Fact Sheet. https://www.who.int/docs/default-source/wpro---documents/countries/china/2018-gats-china-factsheet-cn-en.pdf?sfvrsn=3f4e2da9_2.

[4] Chinese Center for Disease Control and Prevention. 2015 China Adult Tobacco Survey Report. http://www.tcrc.org.cn/UploadFiles/2016-03/318/201603231215175500.pdf.

[5] Chen Z., Peto R., Zhou M., Iona A., Smith M., Yang L., Guo Y., Chen Y., Bian Z., Lancaster G. (2015). Contrasting male and female trends in tobacco-attributed mortality in China: Evidence from successive nationwide prospective cohort studies. Lancet.

[6] Goodchild M., Zheng R. (2019). Tobacco control and healthy China 2030. Tob. Control.

[7] World Health Organization. Healthy China 2030 (from Vision to Action). https://www.who.int/healthpromotion/conferences/9gchp/healthy-china/en/.

[8] Su X., Li L., Griffiths S.M., Gao Y., Lau J.T., Mo P.K. (2015). Smoking behaviors and intentions among adolescents in rural China: The application of the Theory of Planned Behavior and the role of social influence. Addict. Behav..

[9] Li S., Ma C., Xi B. (2016). Tobacco control in China: Still a long way to go. Lancet.

[10] Yang J., Hammond D., Driezen P., O’Connor R.J., Li Q., Yong H.-H., Fong G.T., Jiang Y. (2011). The use of cessation assistance among smokers from China: Findings from the ITC China Survey. Public Health.

[11] National Academies of Sciences Engineering Medicine (2018). Public health consequences of e-cigarettes.

[12] Grana R., Benowitz N., Glantz S.A. (2014). E-cigarettes: A scientific review. Circulation.

[13] Callahan-Lyon P. (2014). Electronic cigarettes: Human health effects. Tob. Control.

[14] Marini S., Buonanno G., Stabile L., Ficco G. (2014). Short-term effects of electronic and tobacco cigarettes on exhaled nitric oxide. Toxicol. Appl. Pharmacol..

[15] Vardavas C.I., Anagnostopoulos N., Kougias M., Evangelopoulou V., Connolly G.N., Behrakis P.K. (2012). Short-term pulmonary effects of using an electronic cigarette: Impact on respiratory flow resistance, impedance, and exhaled nitric oxide. Chest.

[16] Glantz S.A. (2019). The Evidence of Electronic Cigarette Risks Is Catching Up With Public Perception. JAMA Netw. Open.

[17] Abrams D.B., Glasser A.M., Pearson J.L., Villanti A.C., Collins L.K., Niaura R.S. (2018). Harm minimization and tobacco control: Reframing societal views of nicotine use to rapidly save lives. Annu. Rev. Public Health.

[18] Hajek P., Phillips-Waller A., Przulj D., Pesola F., Myers Smith K., Bisal N., Li J., Parrott S., Sasieni P., Dawkins L. (2019). A randomized trial of e-cigarettes versus nicotine-replacement therapy. N. Engl. J. Med..

[19] Johnson L., Ma Y., Fisher S.L., Ramsey A.T., Chen L.-S., Hartz S.M., Culverhouse R.C., Grucza R.A., Saccone N.L., Baker T.B. (2019). E-cigarette usage is associated with increased past-12-month quit attempts and successful smoking cessation in two US population–based surveys. Nicot. Tob. Res..

[20] Berry K.M., Reynolds L.M., Collins J.M., Siegel M.B., Fetterman J.L., Hamburg N.M., Bhatnagar A., Benjamin E.J., Stokes A. (2019). E-cigarette initiation and associated changes in smoking cessation and reduction: The Population Assessment of Tobacco and Health Study, 2013–2015. Tob. Control.

[21] Weaver S.R., Huang J., Pechacek T.F., Heath J.W., Ashley D.L., Eriksen M.P. (2018). Are electronic nicotine delivery systems helping cigarette smokers quit? Evidence from a prospective cohort study of US adult smokers, 2015–2016. PLoS ONE.

[22] Comiford A.L., Rhoades D.A., Spicer P., Dvorak J.D., Ding K., Wagener T.L., Doescher M.P. (2020). Impact of e-cigarette use among a cohort of American Indian cigarette smokers: Associations with cigarette smoking cessation and cigarette consumption. Tob. Control.

[23] Khoudigian S., Devji T., Lytvyn L., Campbell K., Hopkins R., O’Reilly D. (2016). The efficacy and short-term effects of electronic cigarettes as a method for smoking cessation: A systematic review and a meta-analysis. Int. J. Public Health.

[24] Liu X., Lu W., Liao S., Deng Z., Zhang Z., Liu Y., Lu W. (2018). Efficiency and adverse events of electronic cigarettes: A systematic review and meta-analysis (PRISMA-compliant article). Medicine.

[25] Rahman M.A., Hann N., Wilson A., Mnatzaganian G., Worrall-Carter L. (2015). E-cigarettes and smoking cessation: Evidence from a systematic review and meta-analysis. PLoS ONE.

[26] Berry K.M., Fetterman J.L., Benjamin E.J., Bhatnagar A., Barrington-Trimis J.L., Leventhal A.M., Stokes A. (2019). Association of electronic cigarette use with subsequent initiation of tobacco cigarettes in US youths. JAMA Netw. Open.

[27] Vardavas C.I., Filippidis F.T., Agaku I.T. (2015). Determinants and prevalence of e-cigarette use throughout the European Union: A secondary analysis of 26 566 youth and adults from 27 Countries. Tob. Control.

[28] Cullen K.A., Gentzke A.S., Sawdey M.D., Chang J.T., Anic G.M., Wang T.W., Creamer M.R., Jamal A., Ambrose B.K., King B.A. (2019). E-Cigarette use among youth in the United States, 2019. Jama.

[29] Xiao L., Parascandola M., Wang C., Jiang Y. (2019). Perception and current use of e-cigarettes among youth in China. Nicot. Tob. Res..

[30] Wang X., Zhang X., Xu X., Gao Y. (2019). Perceptions and use of electronic cigarettes among young adults in China. Tob. Induc. Dis..

[31] Wang W., He Z., Feng N., Cai Y. (2019). Electronic cigarette use in China: Awareness, prevalence and regulation. Tob. Induc. Dis..

[32] China: Central Government Bans Sale of E-Cigarettes to Minors. https://perma.cc/C5A6-NK7Z.

[33] Circular on Further Protecting Minors from the Harm of E-cigarettes. https://perma.cc/TU8L-2T6G.

[34] China: Government Tightens Regulation of E-Cigarettes. https://www.loc.gov/law/foreign-news/article/china-government-tightens-regulation-of-e-cigarettes/.

[35] Lv J., Su M., Hong Z., Zhang T., Huang X., Wang B., Li L. (2011). Implementation of the WHO framework convention on tobacco control in mainland China. Tob. Control.

[36] Palipudi K.M., Morton J., Hsia J., Andes L., Asma S., Talley B., Caixeta R.D., Fouad H., Khoury R.N., Ramanandraibe N. (2016). Methodology of the global adult tobacco survey—2008–2010. Glob. Health Promot..

[37] Zhao L., Mbulo L., Palipudi K., Wang J., King B. (2019). Awareness and use of e-cigarettes among urban residents in China. Tob. Induc. Dis..

[38] Koplan J., Redmon P., Duan Y., Duan Z., Wood J. (2015). The role of cities in reducing smoking in China. Ann. Glob. Health.

[39] Global Adult Tobacco Survey Collaborative Group (2011). Tobacco Questions for Surveys: A Subset of Key Questions from the Global Adult Tobacco Survey (GATS).

[40] Kalsbeek W.D., Bowling J.M., Hsia J., Mirza S., Palipudi K.M., Asma S. The Global Adult Tobacco Survey (GATS): Sample design and related methods. Section on Survey Methods, Joint Statistical Meetings, 2010. http://www.asasrms.org/Proceedings/y2010/Files/307559_58832.pdf.

[41] Liang X. (2015). Report of China City Adult Tobacco Survey 2013–2014.

[42] Weaver S.R., Kim H., Glasser A.M., Sutfin E.L., Barrington-Trimis J., Payne T.J., Saddleson M., Loukas A. (2018). Establishing consensus on survey measures for electronic nicotine and non-nicotine delivery system use: Current challenges and considerations for researchers. Addic. Behav..

[43] Peugh J.L., Enders C.K. (2004). Missing data in educational research: A review of reporting practices and suggestions for improvement. Review Edu. Rse..

[44] Jiang N., Ho S.Y., Lam T.H. (2017). Electronic cigarette marketing tactics in mainland China. Tob. Control.

[45] World Health Organization. Tobacco Control in China. https://www.who.int/tobacco/about/partners/bloomberg/chn/en/.

[46] Creamer M.R., Wang T.W., Babb S., Cullen K.A., Day H., Willis G., Jamal A., Neff L. (2019). Tobacco product use and cessation indicators among adults—United States, 2018. MMWR Morb. Mortal. Wkly. Rep..

[47] Nargis N., Zheng R., Xu S.S., Fong G.T., Feng G., Jiang Y., Wang Y., Hu X. (2019). Cigarette Affordability in China, 2006–2015: Findings from International Tobacco Control China Surveys. Int. J. Environ. Res. Public Health.

[48] Hu X., Wang Y., Huang J., Zheng R. (2019). Cigarette Affordability and Cigarette Consumption among Adult and Elderly Chinese Smokers: Evidence from A Longitudinal Study. Int. J. Environ. Res. Public Health.

[49] Qian J. (2016). Tobacco control in China: Institutions, bureaucratic noncompliance and policy ineffectiveness. Chinese Politics as Fragmented Authoritarianism.

[50] Coughlin S.S. (1990). Recall bias in epidemiologic studies. J. Clin. Epidemiol..

[51] Grimm P. (2010). Social desirability bias. Wiley Int. Encycl. Mark..

