# Supplementary material for: Use of Electronic Nicotine Delivery Systems (ENDS) in China: Evidence from Citywide Representative Surveys from Five Chinese Cities in 2018
Source: Int J Environ Res Public Health. 2020 Apr 8;17(7):2541. doi: 10.3390/ijerph17072541 (PMC7177332; doi:10.3390/ijerph17072541)
Supplement: Supplementary file 1 [file ijerph-17-02541-s001.pdf]

*Article*

# Use of Electronic Nicotine Delivery Systems (ENDS) in China: Evidence from Citywide Representative Surveys from Five Chinese Cities in 2018

**Jidong Huang [1,*], Zongshuan Duan [1], Yu Wang [1], Pamela B. Redmon [2] and Michael P. Eriksen [1]**

[1] School of Public Health, Georgia State University, Atlanta, GA 30303, USA; zduan3@student.gsu.edu (Z.D.); ywang145@student.gsu.edu (Y.W.); meriksen@gsu.edu (M.P.E.)
[2] Global Health Institutes, Emory University, Atlanta, GA 30322, USA; pam.redmon@emory.edu
* Correspondence: jhuang17@gsu.edu; Tel.: +1-404-413-9337

Received: 4 March 2020; Accepted: 5 April 2020; Published: date

## Supplementary Materials

Table S1: Awareness, ever use, and past-30-day use of ENDS among adult residents in five Chinese cities in 2018.

Table S2: Adjusted ORs of awareness, ever use, and past-30-day use of ENDS among adult residents in five Chinese cities in 2018.

Table S3: Awareness, ever use, and past-30-day use of ENDS among adult smokers in five Chinese cities in 2018.

Table S4: Adjusted ORs of awareness, ever use, and past-30-day use of ENDS among adult smokers in five Chinese cities in 2018.

**Table S1.** Awareness, ever use, and past-30-day use of Electronic Delivery Nicotine Systems (ENDS) among adult residents in five Chinese cities in 2018.

| **Demographic Characteristics** | **Chengdu (N = 1914)** | | **Chongqing (N = 1829)** | | **Wuhan (N = 2251)** | | **Xiamen (N = 2116)** | | **Xi'an (N = 2123)** | | **Overall (N = 10,233)** | |
|---|---|---|---|---|---|---|---|---|---|---|---|---|
| | **%** | **95% CI** | **%** | **95% CI** | **%** | **95% CI** | **%** | **95% CI** | **%** | **95% CI** | **%** | **95% CI** |
| Awareness of ENDS | | | | | | | | | | | | |
| Total | 55.9 | 48.1–63.7 | 45.0 | 38.6–51.4 | 46.7 | 38.6–54.7 | 49.9 | 42.2–57.5 | 58.7 | 51.9–65.6 | 51.3 | 48.0–54.6 |
| Sex | | | | | | | | | | | | |
| Male | 64.8 | 57.4–72.1 | 49.1 | 41.5–56.7 | 55.8 | 46.0–65.5 | 59.3 | 50.5–68.2 | 69.4 | 60.3–78.6 | 59.4 | 55.6–63.2 |
| Female | 47.3 | 38.3–56.2 | 40.7 | 33.4–48.1 | 37.3 | 30.7–43.9 | 39.6 | 30.7–48.4 | 47.6 | 39.8–55.5 | 43.0 | 39.5–46.6 |
| Age (Years) | | | | | | | | | | | | |
| 15–24 | 64.5 | 53.3–75.7 | 58.2 | 46.6–69.8 | 68.3 | 57.9–78.7 | 53.4 | 38.6–68.2 | 62.3 | 52.4–72.2 | 61.1 | 55.9–66.2 |
| 25–44 | 61.3 | 51.1–71.6 | 56.4 | 48.8–63.9 | 50.4 | 39.3–61.5 | 59.5 | 52.5–66.4 | 65.6 | 58.5–72.7 | 59.0 | 55.1–62.9 |
| 45–64 | 50.9 | 43.5–58.2 | 34.7 | 28.4–40.9 | 45.3 | 37.8–52.7 | 32.3 | 21.4–43.2 | 54.9 | 45.9–64.0 | 44.0 | 40.4–47.5 |
| 65 and above | 31.1 | 23.5–38.8 | 18.7 | 11.9–25.6 | 22.0 | 15.0–29.0 | 11.3 | 5.0–17.6 | 32.1 | 23.6–40.6 | 24.5 | 21.1–27.9 |
| Education Level | | | | | | | | | | | | |
| Primary school completed or below | 29.5 | 19.3–39.6 | 12.7 | 8.6–16.9 | 20.3 | 13.3–27.4 | 14.2 | 9.7–18.6 | 43.2 | 26.9–59.5 | 21.2 | 16.9–25.4 |
| Junior high school completed | 44.9 | 38.4–51.5 | 30.3 | 24.6–35.9 | 42.6 | 32.2–53.0 | 45.3 | 36.4–54.1 | 51.5 | 43.4–59.7 | 41.0 | 37.4–44.7 |
| Senior high school completed | 60.4 | 50.3–70.4 | 49.8 | 40.1–59.6 | 50.3 | 40.4–60.2 | 55.6 | 44.5–66.7 | 54.7 | 44.9–64.5 | 53.8 | 49.4–58.1 |
| College degree or above | 63.4 | 53.9–72.9 | 61.6 | 53.0–70.2 | 52.0 | 42.2–61.7 | 63.6 | 54.8–72.3 | 64.6 | 55.2–74.1 | 61.6 | 57.4–65.9 |
| Occupation | | | | | | | | | | | | |
| Gov. employee, teacher, healthcare provider | 63.5 | 51.3–75.8 | 65.2 | 55.4–75.1 | 47.5 | 35.9–59.1 | 37.6 | 24.8–50.5 | 60.8 | 52.0–69.6 | 58.6 | 53.3–63.9 |
| Factory, business, service industry employee | 63.4 | 54.6–72.2 | 46.7 | 39.2–54.2 | 55.9 | 46.2–65.6 | 60.6 | 52.1–69.2 | 70.3 | 63.2–77.3 | 59.8 | 56.0–63.6 |
| Not in the labor force [1] | 46.8 | 39.1–54.5 | 40.7 | 32.7–48.6 | 39.6 | 31.8–47.4 | 39.3 | 32.2–46.4 | 51.2 | 43.2–59.1 | 43.9 | 40.3–47.5 |
| Current Smoking Status | | | | | | | | | | | | |
| Yes | 75.5 | 66.8–84.1 | 56.0 | 46.0–66.1 | 65.3 | 56.5–74.1 | 65.2 | 56.3–74.1 | 78.9 | 71.2–86.5 | 67.8 | 63.7–72.0 |
| No | 50.1 | 42.5–57.8 | 41.5 | 35.2–47.8 | 41.3 | 33.3–49.2 | 45.2 | 37.5–52.9 | 52.7 | 45.3–60.2 | 46.3 | 43.1–49.6 |
| Ever Use of ENDS | | | | | | | | | | | | |
| Total | 4.6 | 3.2–6.0 | 4.4 | 2.9–5.9 | 3.7 | 2.7–4.8 | 4.3 | 2.9–5.6 | 6.6 | 4.5–8.7 | 4.8 | 4.1–5.5 |
| Sex | | | | | | | | | | | | |
| Male | 8.0 | 5.5–10.6 | 7.2 | 4.1–10.3 | 7.2 | 5.7–8.7 | 7.6 | 5.0–10.3 | 12.4 | 8.7–16.2 | 8.6 | 7.2–9.9 |
| Female | 1.3 | 0.4–2.2 | 1.4 | 0.0–2.92 | 0.2 | 0.0–0.5 | 0.6 | 0.0–1.4 | 0.6 | 0.0–1.2 | 0.9 | 0.5–1.4 |

| | | | | | | | | | | | | |
|---|---|---|---|---|---|---|---|---|---|---|---|---|
| Age (Years) | | | | | | | | | | | | |
| 15–24 | 3.6 | 0.9–6.2 | 5.7 | 1.4–10.0 | 6.1 | 3.4–8.7 | 5.5 | 1.0–10.1 | 7.2 | 2.6–11.8 | 5.6 | 3.8–7.5 |
| 25–44 | 5.7 | 3.4–7.9 | 5.0 | 2.1–8.0 | 4.6 | 2.7–6.5 | 4.9 | 3.4–6.4 | 8.0 | 4.7–11.2 | 5.7 | 4.6–6.8 |
| 45–64 | 4.7 | 2.9–6.5 | 3.9 | 1.6–6.2 | 3.1 | 1.9–4.4 | 2.3 | 1.0–3.5 | 6.0 | 4.1–8.0 | 4.2 | 3.3–5.1 |
| 65 and above | 2.2 | 0.3–4.0 | 2.1 | 0.5–3.6 | 0.9 | 0.0–1.9 | 0.5 | 0.0–1.4 | 1.4 | 0.1–2.7 | 1.6 | 0.9–2.3 |
| Education Level | | | | | | | | | | | | |
| Primary school completed or below | 0.4 | 0.0–1.3 | 1.8 | 0.0–3.7 | 1.3 | 0.0–3.0 | 0.3 | 0.0–0.7 | 2.4 | 0.0–5.2 | 1.2 | 0.4–2.0 |
| Junior high school completed | 4.5 | 2.7–6.4 | 4.1 | 1.0–7.1 | 4.0 | 1.1–7.0 | 4.2 | 1.1–7.3 | 6.4 | 2.9–10.0 | 4.5 | 3.2–5.8 |
| Senior high school completed | 4.7 | 2.5–6.8 | 4.4 | 1.4–7.3 | 3.4 | 2.0–4.7 | 7.1 | 4.3–10.0 | 4.9 | 2.5–7.4 | 4.7 | 3.6–5.7 |
| College degree or above | 5.4 | 3.2–7.7 | 5.6 | 3.2–8.1 | 4.5 | 2.6–6.3 | 3.9 | 1.9–6.0 | 8.2 | 5.3–11.1 | 5.9 | 4.8–7.0 |
| Occupation | | | | | | | | | | | | |
| Gov. employee, teacher, healthcare provider | 4.6 | 1.8–7.5 | 3.7 | 1.0–6.4 | 2.8 | 0.1–5.5 | 7.2 | 2.2–12.2 | 3.4 | 0.1–6.8 | 4.1 | 2.7–5.5 |
| Factory, business, service industry employee | 6.2 | 3.6–8.7 | 4.8 | 1.7–7.8 | 5.5 | 3.8–7.3 | 5.1 | 3.1–7.0 | 9.6 | 5.6–13.6 | 6.3 | 5.0–7. |
| Not in the labor force [1] | 3.1 | 1.4–4.8 | 4.3 | 2.9–5.8 | 2.6 | 1.7–3.5 | 2.6 | 1.3–4.0 | 5.1 | 2.7–7.5 | 3.8 | 3.0–4.6 |
| Current Smoking Status | | | | | | | | | | | | |
| Yes | 17.2 | 12.3–22.2 | 14.7 | 8.7–20.6 | 14.4 | 11.1–17.8 | 15.5 | 10.7–20.4 | 23.1 | 15.9–30.3 | 17.1 | 14.5–19.7 |
| No | 0.9 | 0.2–1.7 | 1.0 | 0.2–1.8 | 0.6 | 0.1–1.2 | 0.9 | 0.2–1.6 | 1.7 | 0.4–3.0 | 1.1 | 0.7–1.5 |
| Past-30-Day Use of ENDS | | | | | | | | | | | | |
| Total | 0.7 | 0.3–1.2 | 0.6 | 0.1–1.0 | 1.0 | 0.5–1.4 | 0.9 | 0.4–1.4 | 1.7 | 0.2–3.1 | 0.9 | 0.6–1.3 |
| Sex | | | | | | | | | | | | |
| Male | 1.1 | 0.3–1.9 | 0.7 | 0.0–1.4 | 1.8 | 0.8–2.7 | 1.6 | 0.7–2.6 | 2.9 | 0.6–5.3 | 1.6 | 1.0–2.2 |
| Female | 0.3 | 0.0–1.0 | 0.4 | 0.0–1.0 | 0.2 | 0.0–0.5 | 0.0 | 0.0–0.1 | 0.3 | 0.0–0.7 | 0.3 | 0.1–0.5 |
| Age (Years) | | | | | | | | | | | | |
| 15–24 | 0.4 | 0.0–1.4 | 1.4 | 0.0–3.2 | 1.0 | 0.0–2.5 | 1.0 | 0.0–2.3 | 2.8 | 0.0–6.2 | 1.5 | 0.4–2.5 |
| 25–44 | 1.2 | 0.3–2.1 | 0.1 | 0.0–0.2 | 1.6 | 0.7–2.5 | 0.9 | 0.2–1.6 | 2.0 | 0.0–4.3 | 1.1 | 0.6–1.6 |
| 45–64 | 0.5 | 0.0–1.2 | 0.8 | 0.0–1.7 | 0.4 | 0.0–0.9 | 0.8 | 0.0–1.7 | 0.6 | 0.1–1.1 | 0.6 | 0.3–1.0 |
| 65 and above | 0.0 | | 0.0 | | 0.7 | 0.0–1.6 | 0.0 | | 0.2 | 0.0–0.5 | 0.2 | 0.0–0.4 |
| Education Level | | | | | | | | | | | | |
| Primary school completed or below | 0.0 | | 0.0 | | 0.0 | | 0.1 | 0.0–0.2 | 0.6 | 0.0–1.9 | 0.1 | 0.0–0.2 |
| Junior high school completed | 0.5 | 0.0–1.1 | 0.7 | 0.0–1.8 | 0.5 | 0.0–1.2 | 0.7 | 0.0–1.5 | 1.2 | 0.0–2.9 | 0.7 | 0.2–1.2 |

| | | | | | | | | | | | | |
|---|---|---|---|---|---|---|---|---|---|---|---|---|
| Senior high school completed | 1.4 | 0.0–2.9 | 1.3 | 0.1–2.4 | 0.9 | 0.1–1.7 | 0.8 | 0.1–1.6 | 0.5 | 0.0–1.1 | 1.0 | 0.5–1.4 |
| College degree or above | 0.7 | 0.0–1.4 | 0.2 | 0.0–0.4 | 1.5 | 0.6–2.5 | 1.3 | 0.2–2.5 | 2.6 | 0.0–5.2 | 1.3 | 0.6–1.9 |
| Occupation | | | | | | | | | | | | |
| Gov. employee, teacher, healthcare provider | 0.5 | 0.0–1.2 | 0.1 | 0.0–0.4 | 0.7 | 0.0–1.6 | 1.3 | 0.0–2.7 | 1.2 | 0.0–2.7 | 0.6 | 0.2–1.0 |
| Factory, business, service industry employee | 1.5 | 0.4–2.5 | 0.5 | 0.0–1.3 | 1.4 | 0.1–2.6 | 1.2 | 0.3–2.0 | 2.1 | 0.1–4.1 | 1.3 | 0.8–1.9 |
| Not in the labor force [1] | 0.1 | 0.0–0.3 | 0.7 | 0.0–1.4 | 0.8 | 0.3–1.2 | 0.4 | 0.0–1.0 | 1.5 | 0.0–3.2 | 0.7 | 0.3–1.2 |
| Current Smoking Status | | | | | | | | | | | | |
| Yes | 3.2 | 1.2–5.3 | 2.4 | 0.6–4.2 | 4.0 | 2.0–6.1 | 3.3 | 1.3–5.2 | 6.9 | 1.7–12.2 | 3.9 | 2.6–5.3 |
| No | 0.0 | | 0.0 | | 0.1 | 0.0–0.3 | 0.1 | 0.0–0.3 | 0.1 | 0.0–0.2 | 0.0 | 0.0–0.1 |

[1] Respondents who were not in the labor force included students, homemakers, retired, and unemployed residents either able or unable to work.

**Table S2.** Adjusted[1] ORs of awareness, ever use, and past-30-day use of ENDS among adult residents in five Chinese cities in 2018.

| Indicators | Chengdu | | Chongqing | | Wuhan | | Xiamen | | Xi'an | | Overall | |
|---|---|---|---|---|---|---|---|---|---|---|---|---|
| | OR | 95% CI | OR | 95% CI | OR | 95% CI | OR | 95% CI | OR | 95% CI | OR | 95% CI |
| Awareness | | | | | | | | | | | | |
| *Sex* | | | | | | | | | | | | |
| Male | 1.4 | 0.9–2.1 | 1.0 | 0.7–1.5 | 1.5 | 1.1–2.0 | 1.6 | 1.0–2.6 | 1.7 | 1.0–2.7 | 1.4 | 1.1–1.6 |
| Female | Ref. | | Ref. | | Ref. | | Ref. | | Ref. | | Ref. | |
| *Age Group* | | | | | | | | | | | | |
| 15–24 | 2.4 | 1.4–4.2 | 2.8 | 1.4–5.3 | 5.7 | 2.9–11.6 | 4.5 | 1.7–11.9 | 3.1 | 1.5–6.4 | 2.9 | 2.1–3.8 |
| 25–34 | 1.9 | 1.2–3.1 | 2.9 | 1.7–4.9 | 2.4 | 1.3–4.3 | 4.9 | 2.3–10.3 | 2.8 | 1.7–4.6 | 2.4 | 1.9–3.0 |
| 35–64 | 1.6 | 1.1–2.3 | 1.8 | 1.0–3.2 | 2.2 | 1.4–3.4 | 2.8 | 1.5–5.2 | 2.0 | 1.3–2.9 | 1.7 | 1.4–2.1 |
| 65 and above | Ref. | | Ref. | | Ref. | | Ref. | | Ref. | | Ref. | |
| *Education* | | | | | | | | | | | | |
| Primary school completed or below | Ref. | | Ref. | | Ref. | | Ref. | | Ref. | | Ref. | |
| Junior high school completed | 1.5 | 1.0–2.2 | 2.4 | 1.7–3.5 | 1.9 | 1.3–2.8 | 2.8 | 1.7–4.6 | 1.0 | 0.5–1.9 | 2.0 | 1.6–2.5 |
| Senior high school completed | 2.5 | 1.5–4.0 | 5.0 | 3.0–8.4 | 2.1 | 1.3–3.5 | 4.8 | 2.7–8.6 | 0.8 | 0.4–1.8 | 2.9 | 2.2–3.9 |
| College degree or above | 2.6 | 1.5–4.6 | 7.2 | 4.4–11.8 | 2.3 | 1.3–4.1 | 6.7 | 3.5–13.0 | 1.2 | 0.5–2.8 | 3.9 | 2.8–5.3 |
| *Occupation* | | | | | | | | | | | | |
| Gov. employee, teacher, healthcare provider | 1.3 | 0.9–2.1 | 1.3 | 0.9–2.0 | 1.2 | 0.7–1.8 | 0.8 | 0.5–1.4 | 1.2 | 0.8–1.9 | 1.2 | 1.0–1.5 |
| Factory, business, service industry employee | 1.3 | 0.9–1.8 | 0.9 | 0.6–1.2 | 1.4 | 1.0–2.0 | 1.4 | 0.9–2.3 | 1.7 | 1.1–2.6 | 1.3 | 1.1–1.5 |
| Not in the labor force | Ref. | | Ref. | | Ref. | | Ref. | | Ref. | | Ref. | |
| *Current smoking status* | | | | | | | | | | | | |
| Currently smoking | 2.5 | 1.6–4.0 | 2.0 | 1.4–2.8 | 2.4 | 1.9–3.1 | 2.6 | 1.6–4.1 | 2.5 | 1.7–3.6 | 2.3 | 1.9–2.7 |

| | | | | | | | | | | | | |
|---|---|---|---|---|---|---|---|---|---|---|---|---|
| Currently not smoking | Ref. | | Ref. | | Ref. | | Ref. | | Ref. | | Ref. | |
| Ever Use | | | | | | | | | | | | |
| *Sex* | | | | | | | | | | | | |
| Male | 1.4 | 0.4–4.9 | 1.0 | 0.2–4.6 | 9.1 | 1.4–60.1 | 2.4 | 0.5–10.2 | 6.2 | 1.8–21.9 | 2.2 | 1.1–4.4 |
| Female | Ref. | | Ref. | | Ref. | | Ref. | | Ref. | | Ref. | |
| *Age Group* | | | | | | | | | | | | |
| 15–24 | 0.8 | 0.2–3.7 | 2.3 | 0.6–9.7 | 8.9 | 3.0–26.8 | 7.9 | 0.9–72.3 | 4.8 | 1.4–16.9 | 2.8 | 1.5–5.5 |
| 25–34 | 0.9 | 0.3–3.0 | 1.3 | 0.3–4.7 | 3.7 | 1.2–11.1 | 3.6 | 0.4–37.4 | 3.1 | 1.1–8.5 | 1.7 | 1.0–3.0 |
| 35–64 | 0.8 | 0.3–2.1 | 1.3 | 0.3–4.8 | 2.2 | 0.7–7.2 | 2.1 | 0.2–25.9 | 2.0 | 0.8–5.3 | 1.3 | 0.7–2.4 |
| 65 and above | Ref. | | Ref. | | Ref. | | Ref. | | Ref. | | Ref. | |
| *Education* | | | | | | | | | | | | |
| Primary school completed or below | Ref. | | Ref. | | Ref. | | Ref. | | Ref. | | Ref. | |
| Junior high school completed | 9.8 | 0.9–107.8 | 1.7 | 0.4–6.7 | 1.5 | 0.3–8.2 | 11.5 | 1.4–93.8 | 1.8 | 0.6–6.2 | 2.8 | 1.3–6.0 |
| Senior high school completed | 8.1 | 0.9–76.1 | 1.5 | 0.4–6.0 | 0.8 | 0.2–4.2 | 31.5 | 6.0–165.6 | 0.8 | 0.2–2.9 | 2.4 | 1.1–5.0 |
| College degree or above | 12.8 | 1.1–150.2 | 3.4 | 0.8–14.6 | 1.4 | 0.3–7.2 | 18.1 | 2.0–160.9 | 2.2 | 0.7–6.9 | 4.4 | 2.1–9.2 |
| *Occupation* | | | | | | | | | | | | |
| Gov. employee, teacher, healthcare provider | 1.0 | 0.4–2.1 | 0.6 | 0.3–1.6 | 1.1 | 0.4–3.1 | 4.2 | 1.2–15.4 | 0.5 | 0.2–1.3 | 0.8 | 0.5–1.2 |
| Factory, business, service industry employee | 1.2 | 0.5–2.9 | 0.9 | 0.4–2.0 | 1.3 | 0.7–2.4 | 1.8 | 0.7–4.9 | 1.2 | 0.6–2.4 | 1.1 | 0.8–1.5 |
| Not in the labor force | Ref. | | Ref. | | Ref. | | Ref. | | Ref. | | Ref. | |
| *Current smoking status* | | | | | | | | | | | | |
| Currently smoking | 19.1 | 5.1–72.1 | 18.9 | 5.8–62.3 | 16.4 | 6.4–41.9 | 19.7 | 8.7–44.4 | 11.2 | 4.7–27.0 | 15.5 | 9.1–26.4 |
| Currently not smoking | Ref. | | Ref. | | Ref. | | Ref. | | Ref. | | Ref. | |
| Past-30-Day Use | | | | | | | | | | | | |
| *Sex* | | | | | | | | | | | | |
| Male | 0.3 | 0.0–2.7 | 0.2 | 0.0–1.3 | 0.5 | 0.13–2.03 | 7.4 | 0.4–133.0 | 0.2 | 0.0–2.1 | 0.4 | 0.2–1.1 |
| Female | Ref. | | Ref. | | Ref. | | Ref. | | Ref. | | Ref. | |
| *Age Group* | | | | | | | | | | | | |
| 15–24 | NA | NA | NA | NA | 0.8 | 0.14–4.77 | NA | NA | 16.4 | 0.9–304.0 | 4.5 | 1.1–18.7 |
| 25–34 | NA | NA | NA | NA | 0.9 | 0.2–4.26 | NA | NA | 4.2 | 0.3–71.8 | 2.1 | 0.6–7.4 |
| 35–64 | NA | NA | NA | NA | 0.3 | 0.03–1.76 | NA | NA | 1.3 | 0.1–20.0 | 1.3 | 0.4–4.7 |
| 65 and above | Ref. | | Ref. | | Ref. | | Ref. | | Ref. | | Ref. | |
| *Education* | | | | | | | | | | | | |
| Primary school completed or below | Ref. | | Ref. | | Ref. | | Ref. | | Ref. | | Ref. | |
| Junior high school completed | NA | NA | NA | NA | NA | NA | 5.4 | 0.3–115.8 | 21.2 | 0.5–949.6 | 6.8 | 1.1–41.6 |
| Senior high school completed | NA | NA | NA | NA | NA | NA | 9.0 | 0.6–137.8 | 2.9 | 0.1–64.3 | 7.5 | 1.2–45.5 |
| College degree or above | NA | NA | NA | NA | NA | NA | 21.8 | 1.4–348.5 | 34.4 | 1.3–922.3 | 14.3 | 2.9–71.5 |
| *Occupation* | | | | | | | | | | | | |
| Gov. employee, teacher, healthcare provider | 4.4 | 0.3–79.8 | 0.1 | 0.0–1.4 | 0.7 | 0.14–2.93 | 1.8 | 0.1–41.8 | 0.8 | 0.2–3.5 | 0.5 | 0.2–1.5 |

| | | | | | | | | | | | | |
|---|---|---|---|---|---|---|---|---|---|---|---|---|
| Factory, business, service industry employee | 8.9 | 0.8–95.2 | 0.6 | 0.1–4.2 | 1.0 | 0.3–3.57 | 1.9 | 0.2–20.2 | 1.1 | 0.5–2.6 | 1.1 | 0.6–2.0 |
| Not in the labor force | Ref. | | Ref. | | Ref. | | Ref. | | Ref. | | Ref. | |
| *Current smoking status* | | | | | | | | | | | | |
| Currently smoking | NA | NA | NA | NA | 92.1 | 17.56–483.21 | 19.1 | 2.6–141.8 | NA | NA | 198.7 | 52.3–754.4 |
| Currently not smoking | Ref. | | Ref. | | Ref. | | Ref. | | Ref. | | Ref. | |

[1] Controlling gender, age, education, occupation, and smoking status.

**Table S3.** Awareness, ever use, and past-30-day use of ENDS among adult smokers in five Chinese cities in 2018.

| **Demographic Characteristics** | **Chengdu (N = 414)** | | **Chongqing (N = 399)** | | **Wuhan (N = 521)** | | **Xiamen (N = 490)** | | **Xi'an (N = 515)** | | **Overall (N = 2339)** | |
|---|---|---|---|---|---|---|---|---|---|---|---|---|
| | **%** | **95% CI** | **%** | **95% CI** | **%** | **95% CI** | **%** | **95% CI** | **%** | **95% CI** | **%** | **95% CI** |
| Awareness of ENDS | | | | | | | | | | | | |
| Total | 75.5 | 67.2–83.7 | 56.0 | 46.5–65.5 | 65.3 | 56.9–73.6 | 65.2 | 56.7–73.7 | 78.9 | 71.6–86.1 | 67.8 | 63.2–72.4 |
| Sex | | | | | | | | | | | | |
| Male | 74.9 | 66.2–83.5 | 55.4 | 45.9–65.0 | 66.0 | 57.5–74.5 | 65.0 | 56.6–73.4 | 78.8 | 71.8–85.8 | 67.6 | 62.9–72.2 |
| Female | 80.8 | 71.8–89.8 | 64.9 | 47.9–82.0 | 50.8 | 30.4–71.1 | 70.6 | 53.5–87.6 | 80.9 | 61.6–100.0 | 71.7 | 64.2–79.1 |
| Age (Years) | | | | | | | | | | | | |
| 15–24 | 91.4 | 83.2–99.6 | 84.8 | 69.9–99.7 | 88.6 | 73.6–100.0 | 76.0 | 50.5–100.0 | 80.6 | 65.1–96.1 | 84.3 | 77.3–91.2 |
| 25–44 | 78.0 | 67.8–88.2 | 61.9 | 51.8–72.1 | 73.3 | 63.1–83.5 | 77.9 | 70.1–85.8 | 87.7 | 80.5–94.9 | 75.3 | 70.2–80.3 |
| 45–64 | 67.7 | 56.0–79.4 | 48.6 | 35.2–62.0 | 61.7 | 52.9–70.5 | 42.9 | 30.9–55.0 | 73.3 | 65.8–80.8 | 59.7 | 53.4–66.0 |
| 65 and above | 63.2 | 47.0–79.4 | 15.9 | 3.7–28.1 | 40.6 | 27.9–53.4 | 21.9 | 7.6–36.1 | 47.1 | 32.3–61.8 | 36.3 | 27.4–45.3 |
| Education Level | | | | | | | | | | | | |
| Primary school completed or below | 69.7 | 55.8–83.7 | 10.2 | 1.1–19.4 | 36.1 | 18.3–53.9 | 29.3 | 17.4–41.1 | 71.2 | 45.6–96.8 | 36.2 | 25.4–47.0 |
| Junior high school completed | 65.2 | 52.4–78.1 | 49.0 | 35.4–62.7 | 64.5 | 49.1–79.9 | 58.5 | 46.4–70.6 | 76.7 | 66.9–86.5 | 60.0 | 52.9–67.0 |
| Senior high school completed | 77.0 | 65.8–88.2 | 63.0 | 50.2–75.8 | 65.8 | 56.2–75.4 | 78.9 | 65.5–92.3 | 71.3 | 58.8–83.8 | 69.9 | 64.3–75.4 |
| College degree or above | 79.9 | 70.2–89.6 | 69.9 | 59.8–80.0 | 73.6 | 63.2–84.0 | 86.5 | 72.9–100.0 | 87.6 | 81.7–93.4 | 79.1 | 74.0–84.2 |
| Occupation | | | | | | | | | | | | |
| Gov. employee, teacher, healthcare provider | 68.8 | 49.6–88.0 | 76.5 | 57.6–95.5 | 62.0 | 41.8–82.1 | 52.7 | 31.2–74.2 | 80.2 | 66.2–94.2 | 70.2 | 60.9–79.4 |
| Factory, business, service industry employee | 80.6 | 71.7–89.6 | 55.5 | 45.7–65.2 | 72.6 | 62.3–82.8 | 73.6 | 64.1–83.1 | 84.9 | 78.6–91.1 | 74.3 | 69.4–79.2 |
| Not in the labor force [1] | 69.6 | 59.6–79.7 | 53.2 | 42.4–64.0 | 59.4 | 50.2–68.5 | 57.1 | 46.1–68.1 | 72.1 | 62.3–81.8 | 61.3 | 55.9–66.6 |
| Ever Use of ENDS | | | | | | | | | | | | |
| Total | 17.2 | 12.5–21.9 | 14.7 | 9.0–20.3 | 14.4 | 11.2–17.6 | 15.5 | 11.0–20.1 | 23.1 | 16.2–29.9 | 17.1 | 14.5–19.6 |
| Sex | | | | | | | | | | | | |
| Male | 16.5 | 12.2–20.9 | 14.1 | 7.9–20.4 | 15.1 | 11.8–18.3 | 15.5 | 10.6–20.4 | 23.6 | 16.2–31.1 | 17.0 | 14.3–19.7 |
| Female | 23.2 | 6.1–40.2 | 22.4 | 2.1–42.7 | 1.7 | 0.0–5.1 | 16.4 | 0.0–38.8 | 12.0 | 0.0–30.9 | 18.0 | 8.4–27.6 |
| Age (Years) | | | | | | | | | | | | |
| 15–24 | 22.9 | 10.3–35.6 | 24.3 | 10.2–38.4 | 41.3 | 25.7–56.8 | 29.5 | 6.0–52.9 | 26.0 | 7.8–44.1 | 26.6 | 18.8–34.4 |

| | | | | | | | | | | | | |
|---|---|---|---|---|---|---|---|---|---|---|---|---|
| 25–44 | 19.6 | 11.9–27.2 | 16.1 | 5.0–27.2 | 18.8 | 15.0–22.7 | 17.6 | 12.5–22.8 | 30.6 | 20.8–40.3 | 20.5 | 16.4–24.6 |
| 45–64 | 13.0 | 7.1–18.8 | 11.4 | 3.3–19.4 | 9.3 | 5.2–13.3 | 6.5 | 2.4–10.5 | 16.3 | 11.1–21.4 | 11.9 | 8.7–15.0 |
| 65 and above | 10.4 | 0.1–20.6 | 8.2 | 0.2–16.3 | 4.4 | 0.0–9.1 | 1.8 | 0.0–5.4 | 5.9 | 0.0–12.2 | 6.6 | 3.1–10.1 |
| Education Level | | | | | | | | | | | | |
| Primary school completed or below | 2.5 | 0.0–7.8 | 3.8 | 0.0–9.3 | 6.2 | 0.0–13.4 | 0.9 | 0.0–2.4 | 6.8 | 0.0–15.9 | 3.6 | 0.7–6.5 |
| Junior high school completed | 13.9 | 7.6–20.2 | 12.7 | 2.8–22.7 | 12.8 | 5.0–20.6 | 11.3 | 4.3–18.2 | 24.9 | 11.7–38.0 | 14.3 | 9.8–18.8 |
| Senior high school completed | 15.3 | 9.0–21.5 | 11.9 | 6.6–17.1 | 11.2 | 7.1–15.3 | 22.1 | 11.5–32.8 | 15.5 | 8.0–23.0 | 14.3 | 11.3–17.2 |
| College degree or above | 22.3 | 15.7–28.8 | 23.7 | 14.5–33.0 | 23.5 | 14.7–32.3 | 25.6 | 14.1–37.2 | 31.9 | 20.8–43.0 | 25.5 | 21.1–30.0 |
| Occupation | | | | | | | | | | | | |
| Gov. employee, teacher, healthcare provider | 19.8 | 9.0–30.6 | 13.2 | 4.4–22.0 | 13.3 | 4.6–22.1 | 21.0 | 6.7–35.4 | 11.9 | 2.2–21.6 | 15.7 | 10.6–20.9 |
| Factory, business, service industry employee | 18.8 | 12.6–25.1 | 14.8 | 5.1–24.5 | 19.0 | 14.2–23.8 | 18.2 | 12.0–24.3 | 25.3 | 14.8–35.9 | 19.4 | 15.8–23.0 |
| Not in the labor force [1] | 13.9 | 7.2–20.7 | 14.7 | 9.4–20.0 | 10.7 | 7.0–14.3 | 9.9 | 5.1–14.7 | 22.4 | 15.9–29.0 | 14.9 | 12.1–17.7 |
| Past-30-Day Use of ENDS | | | | | | | | | | | | |
| Total | 3.2 | 1.3–5.2 | 2.4 | 0.6–4.1 | 4.0 | 2.1–6.0 | 3.3 | 1.4–5.1 | 6.9 | 2.0–11.9 | 3.9 | 2.6–5.3 |
| Sex | | | | | | | | | | | | |
| Male | 2.8 | 1.0–4.6 | 1.6 | 0.1–3.2 | 4.2 | 2.1–6.2 | 3.3 | 1.4–5.1 | 6.8 | 2.2–11.5 | 3.6 | 2.3–5.0 |
| Female | 7.6 | 0.0–20.3 | 12.5 | 0.0–27.1 | 1.7 | 0.0–5.1 | 2.8 | 0.0–8.6 | 9.6 | 0.0–26.8 | 8.3 | 1.3–15.3 |
| Age (Years) | | | | | | | | | | | | |
| 15–24 | 2.8 | 0.0–8.5 | 8.3 | 0.0–18.0 | 3.7 | 0.0–10.7 | 7.3 | 0.0–16.0 | 16.6 | 0.0–33.9 | 9.0 | 2.8–15.1 |
| 25–44 | 4.8 | 1.3–8.3 | 0.3 | 0.0–0.9 | 7.5 | 3.1–11.8 | 2.8 | 0.5–5.0 | 8.0 | 1.0–15.0 | 4.4 | 2.5–6.4 |
| 45–64 | 1.9 | 0.0–4.1 | 2.8 | 0.0–5.8 | 1.4 | 0.0–3.1 | 2.6 | 0.0–5.6 | 1.8 | 0.5–3.1 | 2.1 | 1.0–3.2 |
| 65 and above | NA | NA | NA | NA | 3.8 | 0.0–8.3 | NA | NA | 1.2 | 0.0–3.6 | 1.2 | 0.0–2.5 |
| Education Level | | | | | | | | | | | | |
| Primary school completed or below | NA | NA | NA | NA | NA | NA | 0.3 | 0.0–0.9 | 2.6 | 0.0–7.0 | 0.4 | 0.0–1.1 |
| Junior high school completed | 2.0 | 0.0–4.7 | 2.5 | 0.0–6.4 | 2.4 | 0.0–5.3 | 2.0 | 0.0–4.0 | 5.2 | 0.0–11.7 | 2.6 | 0.8–4.5 |
| Senior high school completed | 4.8 | 0.0–9.6 | 4.6 | 0.5–8.7 | 3.2 | 0.7–5.8 | 2.4 | 0.0–4.9 | 1.7 | 0.0–3.7 | 3.4 | 1.8–5.0 |
| College degree or above | 3.3 | 0.0–6.6 | 0.8 | 0.0–1.9 | 7.9 | 3.2–12.5 | 8.7 | 1.4–16.0 | 13.1 | 2.8–23.4 | 6.3 | 3.1–9.6 |
| Occupation | | | | | | | | | | | | |
| Gov. employee, teacher, healthcare provider | 2.3 | 0.0–5.3 | 0.7 | 0.0–2.2 | 3.8 | 0.0–7.9 | 2.9 | 0.0–6.8 | 3.4 | 0.0–8.7 | 2.4 | 0.6–4.2 |
| Factory, business, service industry employee | 5.2 | 1.6–8.8 | 2.0 | 0.0–4.8 | 4.7 | 0.2–9.2 | 4.9 | 1.6–8.1 | 7.2 | 1.3–13.1 | 4.9 | 3.0–6.8 |
| Not in the labor force [1] | 0.6 | 0.0–1.6 | 3.0 | 0.3–5.7 | 3.5 | 1.5–5.5 | 1.0 | 0.0–2.7 | 7.7 | 0.0–15.4 | 3.4 | 1.4–5.4 |

[1] Respondents who were not in the labor force included students, homemakers, retired, and unemployed residents either able or unable to work.

**Table S4.** Adjusted[1] ORs of awareness, ever use, and past-30-day use of ENDS among adult smokers in five Chinese cities in 2018.

| Indicators | Chengdu | | Chongqing | | Wuhan | | Xiamen | | Xi'an | | Overall | |
|---|---|---|---|---|---|---|---|---|---|---|---|---|
| | OR | 95% CI | OR | 95% CI | OR | 95% CI | OR | 95% CI | OR | 95% CI | OR | 95% CI |
| **Awareness** | | | | | | | | | | | | |

| | | | | | | | | | | | | |
|---|---|---|---|---|---|---|---|---|---|---|---|---|
| *Sex* | | | | | | | | | | | | |
| Male | 0.7 | 0.4–1.4 | 0.7 | 0.3–1.9 | 1.1 | 0.5–2.9 | 0.5 | 0.3–1.1 | 0.7 | 0.2–2.1 | 0.6 | 0.4–1.0 |
| Female | Ref. | | Ref. | | Ref. | | Ref. | | Ref. | | Ref. | |
| *Age Group* | | | | | | | | | | | | |
| 15–24 | 4.7 | 1.2–19.0 | 15.1 | 3.0–76.3 | 7.3 | 1.2–44.1 | 4.6 | 0.7–29.2 | 4.1 | 0.9–18.9 | 5.1 | 2.5–10.5 |
| 25–34 | 1.6 | 0.6–4.3 | 5.0 | 1.5–17.1 | 2.6 | 1.1–6.2 | 6.2 | 1.9–21.1 | 5.1 | 1.5–17.1 | 2.9 | 1.9–4.4 |
| 35–64 | 1.1 | 0.5–2.4 | 4.6 | 1.4–15.0 | 1.8 | 0.9–3.5 | 1.9 | 0.7–5.2 | 2.7 | 1.1–6.3 | 1.9 | 1.3–2.8 |
| 65 and above | Ref. | | Ref. | | Ref. | | Ref. | | Ref. | | Ref. | |
| *Education* | | | | | | | | | | | | |
| Primary school completed or below | Ref. | | Ref. | | Ref. | | Ref. | | Ref. | | Ref. | |
| Junior high school completed | 0.8 | 0.3–1.9 | 6.4 | 2.3–17.9 | 2.1 | 0.8–5.6 | 1.6 | 0.8–3.3 | 1.2 | 0.5–2.9 | 2.2 | 1.5–3.3 |
| Senior high school completed | 1.2 | 0.6–2.6 | 9.8 | 3.1–31.4 | 2.1 | 0.9–4.9 | 4.3 | 1.8–10.1 | 0.6 | 0.2–1.7 | 2.9 | 1.9–4.5 |
| College degree or above | 1.2 | 0.5–3.3 | 12.6 | 3.3–48.3 | 2.4 | 0.8–7.3 | 6.7 | 1.8–25.3 | 1.5 | 0.5–4.8 | 4.2 | 2.7–6.6 |
| *Occupation* | | | | | | | | | | | | |
| Gov. employee, teacher, healthcare provider | 0.7 | 0.3–1.7 | 1.4 | 0.5–3.7 | 0.9 | 0.4–2.2 | 0.9 | 0.3–2.3 | 1.2 | 0.4–3.8 | 1.0 | 0.7–1.6 |
| Factory, business, service industry employee | 1.3 | 0.7–2.7 | 0.9 | 0.5–1.6 | 1.2 | 0.7–2.1 | 1.2 | 0.4–3.1 | 1.6 | 0.9–2.9 | 1.3 | 1.0–1.7 |
| Not in the labor force | Ref. | | Ref. | | Ref. | | Ref. | | Ref. | | Ref. | |
| Ever Use | | | | | | | | | | | | |
| *Sex* | | | | | | | | | | | | |
| Male | 0.6 | 0.2–1.7 | 0.4 | 0.1–2.0 | 8.3 | 0.7–94.7 | 0.9 | 0.1–11.7 | 1.4 | 0.2–8.4 | 0.8 | 0.4–1.6 |
| Female | Ref. | | Ref. | | Ref. | | Ref. | | Ref. | | Ref. | |
| *Age Group* | | | | | | | | | | | | |
| 15–24 | 1.2 | 0.2–6.8 | 2.5 | 0.6–10.9 | 11.7 | 2.9–47.2 | 6.2 | 0.5–71.5 | 6.1 | 1.3–29.4 | 3.3 | 1.6–6.6 |
| 25–34 | 1.1 | 0.3–4.8 | 1.0 | 0.2–5.2 | 3.6 | 1.1–12.2 | 3.4 | 0.4–30.0 | 6.3 | 1.3–29.5 | 2.1 | 1.1–4.0 |
| 35–64 | 0.8 | 0.2–2.9 | 1.3 | 0.3–5.6 | 2.0 | 0.5–7.4 | 1.9 | 0.2–21.7 | 3.2 | 0.8–13.1 | 1.5 | 0.8–2.8 |
| 65 and above | Ref. | | Ref. | | Ref. | | Ref. | | Ref. | | Ref. | |
| *Education* | | | | | | | | | | | | |
| Primary school completed or below | Ref. | | Ref. | | Ref. | | Ref. | | Ref. | | Ref. | |
| Junior high school completed | 6.6 | 0.6–80.0 | 3.4 | 0.7–16.8 | 1.0 | 0.2–6.6 | 10.6 | 1.7–88.3 | 4.4 | 0.9–22.1 | 3.9 | 1.7–9.2 |
| Senior high school completed | 6.9 | 0.7–72.5 | 2.4 | 0.4–13.8 | 0.8 | 0.1–4.3 | 23.6 | 3.7–149.3 | 1.8 | 0.4–8.2 | 3.3 | 1.4–7.9 |
| College degree or above | 10.1 | 0.7–14.4 | 8.8 | 1.6–48.4 | 1.3 | 0.2–7.4 | 21.5 | 2.7–168.2 | 5.0 | 1.3–20.0 | 6.9 | 3.0–15.9 |
| *Occupation* | | | | | | | | | | | | |
| Gov. employee, teacher, healthcare provider | 1.1 | 0.4–2.6 | 0.5 | 0.2–1.2 | 1.1 | 0.4–2.8 | 3.6 | 0.9–15.2 | 0.3 | 0.1–1.0 | 0.7 | 0.5–1.1 |
| Factory, business, service industry employee | 1.1 | 0.4–2.7 | 1.0 | 0.4–2.3 | 1.3 | 0.6–2.5 | 1.7 | 0.6–5.1 | 0.8 | 0.5–1.3 | 1.0 | 0.7–1.4 |
| Not in the labor force | Ref. | | Ref. | | Ref. | | Ref. | | Ref. | | Ref. | |
| Past-30-Day Use | | | | | | | | | | | | |
| *Sex* | | | | | | | | | | | | |
| Male | 0.3 | 0.0–2.7 | 0.2 | 0.0–1.3 | 1.1 | 0.1–9.5 | 1.0 | 0.1–12.9 | 0.2 | 0.0–4.1 | 0.4 | 0.1–1.0 |

| | | | | | | | | | | | | |
|---|---|---|---|---|---|---|---|---|---|---|---|---|
| Female | Ref. | | Ref. | | Ref. | | Ref. | | Ref. | | Ref. | |
| *Age Group* | | | | | | | | | | | | |
| 15–24 | NA | NA | NA | NA | 0.4 | 0.1–3.4 | NA | NA | 16.8 | 0.9–323.9 | 4.4 | 1.1–18.7 |
| 25–34 | NA | NA | NA | NA | 1.1 | 0.2–4.8 | NA | NA | 3.8 | 0.2–67.5 | 2.0 | 0.6–7.0 |
| 35–64 | NA | NA | NA | NA | 0.3 | 0.0–1.8 | NA | NA | 1.3 | 0.1–20.9 | 1.3 | 0.4–4.6 |
| 65 and above | Ref. | | Ref. | | Ref. | | Ref. | | Ref. | | Ref. | |
| *Education* | | | | | | | | | | | | |
| Primary school completed or below | Ref. | | Ref. | | Ref. | | Ref. | | Ref. | | Ref. | |
| Junior high school completed | NA | NA | NA | NA | NA | NA | 4.5 | 0.2–121.8 | NA | NA | 7.0 | 1.1–43.4 |
| Senior high school completed | NA | NA | NA | NA | NA | NA | 5.7 | 0.3–118.9 | 2.8 | 0.1–76.4 | 7.5 | 1.2–46.1 |
| College degree or above | NA | NA | NA | NA | NA | NA | 19.9 | 1.0–381.2 | NA | NA | 14.6 | 2.9–74.2 |
| *Occupation* | | | | | | | | | | | | |
| Gov. employee, teacher, healthcare provider | 4.4 | 0.2–81.8 | 0.1 | 0.0–1.4 | 0.6 | 0.1–2.8 | 1.6 | 0.0–105.3 | 0.5 | 0.1–3.3 | 0.4 | 0.1–1.3 |
| Factory, business, service industry employee | 8.9 | 0.8–97.2 | 0.6 | 0.1–4.3 | 0.9 | 0.2–3.1 | 3.1 | 0.3–37.4 | 1.1 | 0.5–2.7 | 1.1 | 0.6–2.0 |
| Not in the labor force | Ref. | | Ref. | | Ref. | | Ref. | | Ref. | | Ref. | |

[1] Controlling gender, age, education, and occupation.
